# A RATional choice for translational research?

**DOI:** 10.1242/dmm.027706

**Published:** 2016-10-01

**Authors:** Tim Aitman, Paraminder Dhillon, Aron M. Geurts

**Affiliations:** 1 Centre for Genomic and Experimental Medicine, Institute of Genetics and Molecular Medicine, University of Edinburgh, Edinburgh EH4 2XU, UK; 2 Disease Models & Mechanisms, The Company of Biologists, Bidder Building, Station Road, Histon, Cambridge CB24 9LF, UK; 3 Department of Physiology and Genome Editing Rat Resource Center, Medical College of Wisconsin, Milwaukee, WI 53226, USA

**Keywords:** CRISPR, Genomics, Model organism, Rat, Translational

## Abstract

Future prospects continue to be strong for research using the rat as a model organism. New technology has enabled the proliferation of many new transgenic and knockout rat strains, the genomes of more than 40 rat strains have been sequenced, publications using the rat as a model continue to be produced at a steady rate, and discoveries of disease-associated genes and mechanisms from rat experiments abound, frequently with conservation of function between rats and humans. However, advances in genome technology have led to increasing insights into human disease directly from human genetic studies, pulling more and more researchers into the human genetics arena and placing funding for model organisms and their databases under threat. This, therefore, is a pivotal time for rat-based biomedical research – a timely moment to review progress and prospects – providing the inspiration for a new Special Collection focused on the impact of the model on translational science, launched in this issue of Disease Models & Mechanisms. What disease areas are most appropriate for research using rats? Why should the rat be favoured over other model organisms, and should the present levels of funding be continued? Which approaches should we expect to yield biologically and medically useful insights in the coming years? These are key issues that are addressed in the original Research Articles and reviews published in this Special Collection, and in this introductory Editorial. These exemplar articles serve as a landmark for the present status quo after a decade of major advances using the rat model and could help to guide the direction of rat research in the coming decade.

## Abstract (precis)

**Summary:** This Editorial introduces a new Special Collection, ‘Spotlight on Rat: Translational Impact’, providing a summary of the articles published in this launch issue and a flavour of what's to come, as well as highlighting the impact rat models have made and continue to make on understanding human disease.

## Putting the ‘rat’ in laboratory

**Fig. 1.**
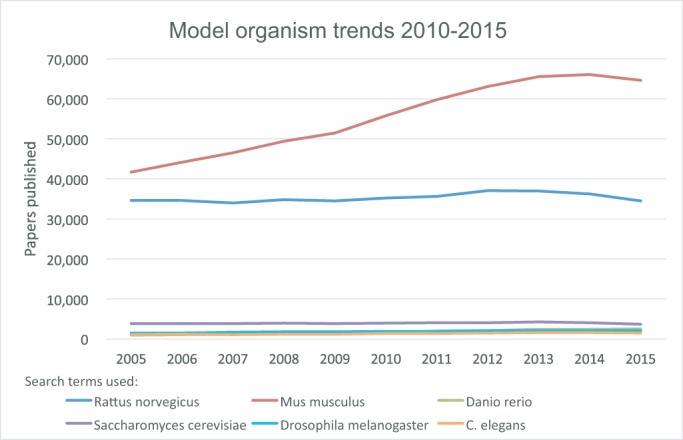
**Trends in model organism publications (2005-2015).** Model organism papers published between 2005 and 2015, as revealed by querying National Center for Biotechnology Information (NCBI) PubMed using the indicated species as search terms.

The rat is one of the premier models for studies of physiology, pharmacology, toxicology and neurobehaviour, reflected by an immense literature heritage. Over 1.5 million PubMed entries include the terms ‘Rattus norvegicus’; almost as many as for all other model organisms combined. Although there have been more publications on the mouse in the last decade, publications using the rat model continue to be strong (Fig. 1) and, as foretold in an Editorial introducing a *Nature Genetics* issue focused on rat genetics in 2008, “Although the mouse is still the mammalian genetic model of choice, the gap may be closing” (Editorial, 2008). Eight years on from those heady days, how does the landscape lie?

One way of assessing the strength of a model system is to consider the techniques and infrastructure available for its study. Another measure of a model's ‘success’, particularly relevant to studies of human disease, is its applicability to human physiology and pathophysiology. Relevant to the availability of tools, the first rat genome was sequenced in 2004 – it was the third mammalian genome sequence ever to have been completed. Over 40 rat genomes have been sequenced since that time, including 27 genomes for which an evolutionary analysis defined the phylogenetic history and selection pressures underlying derivation of 11 widely used models of hypertension and diabetes (Atanur et al., 2013). Although initially lagging behind the mouse and other model organisms in terms of gene-targeting technologies, the rat was the first mammal in which zinc-finger nucleases (ZFNs) were used by embryo microinjection to create a gene knockout (Geurts et al*.*, 2009). Since then, in just one centre alone – the Genome Editing Rat Resource Center (GERRC) at the Medical College of Wisconsin (MCW) – around 200 rat gene knockout, knock-in, conditional, transgenic and Cre-recombinase driver lines have been created or are at various stages of development for distribution to the research community (http://rgd.mcw.edu/rgdweb/models/gerrc.html). Over 200 additional transgenic or gene mutants have been created in other centres, including 15 in the European Union (EU)-funded ELABORATE programme based in Edinburgh. Two major rat model repositories, the Rat Resource & Research Center (http://www.rrrc.us) in Missouri and the National Bio Resource Project – Rat in Kyoto, Japan (http://www.anim.med.kyoto-u.ac.jp/NBR/) provide the community with options for long-term preservation and distribution of these models.

Without doubt, the rat remains a key model for basic and preclinical studies of pharmacology and toxicology, underlining its importance in studies of human disease. A PubMed search using Medical Subject Headings (MeSH) terms (Pharmacology) OR (Toxicology) AND rat, mouse or other model organisms, revealed 6230 articles for rat, 7495 for mouse and less than 1000 combined for fly, zebrafish, worm and yeast, between January 2000 and August 2016. For physiological studies, the rat has the advantage of size – and therefore ease and precision of measurements – over the mouse, although higher maintenance costs for rats and the opportunities of probe miniaturisation and imaging have offset these advantages to some extent in recent years. For neurobehaviour, compared to other model systems, the larger forebrain size and greater sophistication and sociability of the rat continue to provide more power and comparability with humans (Ellenbroek and Youn, 2016).

However, research funding is increasingly competitive for model organism communities. Throughout the 1990s and 2000s, major funds provided by the National Institutes of Health (NIH) and the EU drove the sequencing of the first rat genome and, supported by the EU consortia EURATools and EURATRANS, the development of genome tools and databases led to a host of gene discoveries, many of which were translated to human disease (Aitman et al., 2008). Although the discovery of disease genes and mechanisms has continued, the rat community has been under increasing pressure in recent years. No concerted EU-derived funding of rat research followed the EURATRANS programme, and NIH funding for model organism databases, including the outstanding Rat Genome Database (RGD; described below), which is funded only until 2018, is potentially under threat. However, a recent petition in support of model organism databases gained over 11,000 signatures and generated a positive response from the NIH that recognises the need for continuing support. More recently, global uncertainty in the rat genetics community resulted in the unexpected cancellation of an international meeting that has been running annually for more than 15 years – the Rat Genomes and Models meeting. This meeting, which rotated between Cold Spring Harbor Laboratory in New York, USA, the Wellcome Trust Sanger Institute in Hinxton, UK, and Kyoto, Japan, represented a key opportunity for networking and collaboration within the rat genetics community. In the Model for Life interview published in this issue, Howard Jacob discusses the impact that the early years of this meeting had on the rat genetics field and beyond (Jacob, 2016).

Notwithstanding these challenges, the rat model research community remains active and enthusiastic, the quality of ongoing research remains high, and publication rates remain steady (Fig. 1). High-profile research groups across the world and strong ongoing programmes for generating rat knockouts (NIH GERRC and EU ELABORATE) take advantage of the genome-editing tools and technologies now in place to use the rat model as a springboard for giving insights into human disease (Zhao et al., 2015; Fernandez-Duenas et al., 2015; Wang et al., 2016). In this issue of Disease Models & Mechanisms (DMM), we are pleased to launch a timely Special Collection entitled *Spotlight on Rat: Translational Impact*, which highlights the strengths and ongoing commitment of researchers and funders to studies of the rat model for generating insights into mechanisms underlying human disease. This issue is fully compiled of articles (research, reviews and others) focused on the use of rats for translational research, and further articles will be added to the collection in upcoming issues of the journal. The collection page (http://dmm.biologists.org/collection/rat-disease-model) also features some of the most impactful rat-based articles published in DMM in recent years.

## Frontrunners: reviews and more

Kicking off the review section of the issue, Howard Jacob, pioneer in the rat genetics community, provides a retrospective view of the field in an exclusive interview. Describing his path from pharmacology student to leader of a clinical genomics centre for paediatric patients, Howard explains why he firmly believes that the rat – as well as other model organisms – has a key role to play in this era of genomic medicine (Jacob, 2016).

Among other contributions to the rat genetics and genomics toolbox during his years at MCW, Howard played a central part in the establishment of RGD in 1999. Since then, the database has evolved into the leading resource to obtain genetic, genomic, phenotype and disease-relevant rat data and to make comparisons with corresponding human and mouse data. In a Special Article also published in this issue, Mary Shimoyama and other curators of RGD describe – with worked examples – how the impressive suite of tools and datasets can be used to leverage rat studies to provide new insights into disease pathways and genetics (Shimoyama et al., 2016).

Despite initially trailing behind the mouse in the availability of tools and technologies for generating recombinant models – a topic that will be discussed in depth by Aron Geurts and Tomoji Mashimo in an upcoming DMM Review article – the rat has always been at the forefront as a model in genome-wide mapping studies. As highlighted by Aida Moreno-Moral and Enrico Petretto in the first of three new Reviews published in this issue, the first fully ‘integrative’ genomics study was performed in rats (Moreno-Moral and Petretto, 2016). The availability of specialised inbred strains and large panels for genetic mapping meant that several additional integrative-genomics (and, more recently, ‘systems-genetics’) studies exploiting the model to link genotype with phenotype then followed. This has resulted in advances in our understanding of the genetics of many complex human conditions, ranging from hypertension to alcohol addiction. Indeed, systems-genetics analysis of rats was used to shed light on diet-induced obesity in a new research study published in this issue (Martínez-Micaelo et al., 2016; described further below).

Gene-mapping strategies in rat models have also led to a better understanding of the genetic underpinnings of rheumatoid arthritis. A second Review in this issue, contributed by Anthony Yau and Rikard Holmdahl, describes arthritis-associated genetic polymorphisms that have been identified by genetic linkage and positional cloning using rat congenic strains (Yau and Holmdahl, 2016). The authors provide examples to illustrate how exploration of arthritis-linked polymorphisms using rats has provided new insights that are relevant to clinical autoimmune arthritis.

A research area in which rats have truly shone as a model system is in neuroscience. Bart Ellenbroek and Jiun Youn provide a broad overview of the utility and advantages of rats in neuroscientific studies in their At a Glance poster article (Ellenbroek and Youn, 2016). The authors discuss how fundamental differences between rats and mice result in substantially different outcomes in a variety of neurobehavioural tests and impact on studies of neurodegeneration. While emphasising the unique advantages and limitations associated with each species, they provide an argument for rats being the better model for exploring neuropsychiatric traits. A related Review published previously in DMM critically appraises rat-based insights into the biological basis of addictive behaviour (Jupp et al., 2013).

The final Review in this issue, by Jacob Kjell and Lars Olson, highlights the tremendous progress that has been made in understanding and treating spinal cord injuries based on rat research (Kjell and Olson, 2016). From enabling a picture of the pathological events involved in different forms of spinal cord injury to be built, to facilitating the evaluation of repair strategies, the rat has provided a key experimental model for this area of research for many years, and is poised to continue to enable bench-to-bedside advances.

## Original research contributions: the uniqueness of the rat model

Also showcased in this issue are ten original research articles that serve as paradigms of the ways in which the rat stands as a distinctive model system across diverse areas, including neuroscience and neurobehaviour, musculoskeletal disease, oncology, metabolism, and infection and immunity.

In the first of four research articles relevant to the neuroscience and neurobehaviour field, Judith Homberg and colleagues studied a rat strain with an N-ethyl-N-nitrosourea (ENU) mutagenesis-induced dysfunctional *Drd1* dopamine receptor with decreased transmembrane insertion (Homberg et al., 2016). The mutant strain displayed a deficit in several aspects of social cognition, providing much-needed clarification of the neurobehavioral function of this receptor where previous analysis of *Drd1* knockout mice had given rise to inconsistent findings. In another contribution from the Ellenbroek group, exposure to lipopolysaccharide (LPS) in pregnant rats was used to mimic the known effects of maternal infection and inflammation that impair fetal brain development, predispose to cognitive deficits and, in humans, increase the risk of schizophrenia. The team tested the hypothesis that a high incidence of smoking in schizophrenics might be due to the putative cognitive-enhancing effects of nicotine. In support of this effect, the authors found that global cognitive deficits induced by early LPS exposure were at least in part reversed by nicotine treatment in the offspring (Waterhouse et al., 2016).

Two new studies combined elements of neuroscience and pharmaco/toxicology. Using a model of excitotoxically induced vestibular dysfunction based on transtympanic injection of kainic acid (a neuroexcitatory molecule), Sophie Gaboyard-Niay and colleagues found that vestibular dysfunction occurred shortly after excitotoxic insult, and that recovery correlated closely with changes in the structure and ultrastructure of vestibular hair cells and afferent nerve terminals (Gaboyard-Niay et al., 2016). This model could provide a useful platform to investigate the mechanisms of spontaneous repair of vestibular afferent terminals and to test regenerative therapies. In the second study, Rong-Kung Tsai and co-authors aimed to determine the therapeutic window and anti-inflammatory mechanism for the neuroprotective effect of granulocyte colony-stimulating factor (G-CSF), using an established rat model of acute ischemic optic neuropathy. They showed that early treatment with G-CSF rescued visual function and retinal ganglion cell survival, most likely through stabilising the blood–brain barrier and reducing macrophage infiltration (Wen et al., 2016).

Emphasising the value of the close synteny of the rat and human genomes, Jayleen Grams and colleagues studied bone biomechanics in an osteocalcin-null rat created using CRISPR/Cas9 gene technology (technique illustrated in Ellenbroek and Youn, 2016). Because the osteocalcin locus has undergone triplication in the mouse genome, the rat, which, like humans, has a single osteocalcin gene, has the potential to be more relevant for studies of human osteocalcin gene variation. Osteocalcin knockout rats demonstrated increased trabecular thickness and improved functional quality under biomechanical stress, suggesting new therapeutic approaches for osteoporosis and osteoarthritis (Lambert et al., 2016).

A new *Tp53* mutant rat, created by transferring a mutant *Tp53* allele from an outbred genetic background to the inbred F344 strain, is described in this issue by Elizabeth Bryda, James Amos-Landgraf and colleagues (Hansen et al., 2016). The team report that the mutant shows a distinctive tumour spectrum, the primary tumour types being osteosarcomas and meningeal sarcomas, similar to the early bone and central nervous system (CNS) sarcomas found in humans with *Tp53* mutations, but not commonly found in mouse *Tp53* mutants. Such commonality with human cancer phenotypes has previously been seen in the *Apc* mutant Pirc rat, with intestinal tumour distribution more typical of human intestinal tumours than *Apc* mutant mice (Irving et al., 2014), and in the hormonal responsiveness of rat breast cancer models (Samuelson et al., 2007). This study gives further credence to the translational validity of rat models of human cancer and fills an important gap by providing a new animal model for the study of paediatric osteosarcomas.

Rats have also been widely used in studies of metabolic disease, not least because of their many physiological parallels with humans. In a systems-genetics study of peripheral blood monocytes in rats on a cafeteria-fed diet, Jacques Behmoaras and colleagues identified a cluster of genes – with the acyl-CoA synthetase gene *Acss2* at its hub – that underlies differences between rat strains in their metabolic responses to obesity (Martínez-Micaelo et al., 2016). Furthermore, Susan Ozanne et al. identified and characterised an accelerated aging and oxidative-stress phenotype in the skeletal muscle of offspring of low-protein-fed dams, suggesting that postnatal antioxidant intervention could reduce the increased risk of diabetes and cardiovascular disease in individuals exposed *in utero* to maternal malnutrition (Tarry-Adkins et al., 2016). Earlier this year, the Ozanne lab used rats to provide evidence that a serotonin-mediated mechanism underlies the developmental programming of obesity (Martin-Gronert et al., 2016).

The biology of hepatitis E infection is poorly understood, warranting the generation and characterisation of robust animal models of the infection. Now, Johan Neyts and colleagues have developed a new model of hepatitis E infection in the athymic nude rat (Debing et al., 2016). Given the paucity of models in other species and the increasing recognition of problems with hepatitis E infection in transplantation, this model is likely to prove valuable for further understanding of hepatitis E infection and antiviral studies. Finally, Robert Weissert and colleagues studied gene expression from CNS-infiltrating cells in the well-established myelin-oligodendrocyte-glycoprotein-induced experimental autoimmune encephalomyelitis (EAE) rat model of multiple sclerosis (Herrmann et al., 2016). The conclusion that *Cd38* is involved in the development of EAE in this model was supported by comparative experiments in the *Cd38* knockout mouse.

## Concluding remarks

As demonstrated by the research and reviews published in this issue, and the hugely positive response to our ‘call for papers’, with submissions coming in from excellent groups around the world, the rat model continues to be a leading model for studies of physiology, pharmacology, toxicology and neuroscience. The reasons for this are clear – the close evolutionary and genomic relationship to humans, the sophistication and sociability of the species, the ease of physiological and behavioural measurements, the huge heritage collection of genetic, genomic and other biological data, and the continuing strong publications of important findings relevant to the understanding of human disease. Notwithstanding the pull of human genetics and the struggle for concerted funding for community activities, including meetings and databases, this Special Collection highlights that rat research is driven by a forward-looking community, dedicated to increasing our understanding of basic biology and human disease.

Traditional quantitative trait locus studies, with the need for lengthy fine-mapping and testing of candidate genes, continue to give valuable insights (for example Zhao et al., 2015; Fernandez-Duenas et al., 2015; Wang et al., 2016, and reviewed in Yau and Holmdahl, 2016), but, as with research using other model organisms, these approaches are being replaced by gene targeting using CRISPR/Cas9 and other techniques, as exemplified by the GERRC programme (rgd.mcw.edu/wg/gerrc) and the osteocalcin study in this issue (Lambert et al., 2016). These programmes use gene targeting to knock out genes implicated in the aetiology of common human diseases via genome-wide association studies (GWAS) or other patient-based studies, and allow analysis of gene function that will give insights into disease pathogenesis.

The present challenges for cohesiveness of the rat genetics community stand to be overcome by the substantial body of researchers who recognise the natural advantages of the rat as a model of human disease, as outlined above, and as a model system for testing new drug therapies. The underlying paradigm, that strategic advances in disease prevention and therapy most naturally arise from a deep understanding of disease pathogenesis, make it likely that the rat model will remain one of the two most widely used species for biomedical and pharmaceutical research in the next decade and beyond.
